# Considerações metodológicas sobre o estudo das ondas de calor extremo e mortalidade cardiovascular no Sudeste do Brasil

**DOI:** 10.1590/0102-311XPT210326

**Published:** 2026-10-30

**Authors:** Fábio Luiz Vieira

**Affiliations:** 1 My Brazilian Doctor, São Paulo, Brasil.

Prezadas Editoras,

As ondas de calor extremo representam um dos principais desafios atuais para a saúde pública, e estudos que avaliam seus efeitos sobre a mortalidade são fundamentais para subsidiar políticas de adaptação às mudanças climáticas. Nesse contexto, lemos com interesse o artigo de Almeida et al. ^1^, que aplica o *Excess Heat Factor* (EHF; Fator de Calor Excessivo) de Nairn & Fawcett ^2^ à caracterização de eventos térmicos extremos em metrópoles brasileiras. A transposição desse índice para o contexto do Sudeste brasileiro é uma contribuição relevante, e registramos que os autores declaram explicitamente, no primeiro parágrafo da seção *Métodos*, que o desenho adotado não permite inferência causal. Ao examinar a metodologia descrita, entretanto, identificamos cinco pontos que nos parecem merecer esclarecimento, por poderem influenciar a interpretação e a reprodutibilidade do trabalho.

## Número de óbitos analisados

Os autores informam ter analisado 9.357.684 óbitos, obtidos por local de residência e restritos às doenças do aparelho circulatório (10ª revisão da Classificação Internacional de Doenças - CID-10, Capítulo IX), em todos os municípios das quatro regiões metropolitanas estudadas, entre 2014 e 2023.

Consulta ao Sistema de Informações sobre Mortalidade (SIM) para o mesmo período, igualmente por local de residência, mostra valores substancialmente menores. Os quatro estados correspondentes - São Paulo, Rio de Janeiro, Minas Gerais e Espírito Santo - registraram 1.705.090 óbitos por doenças do aparelho circulatório e 6.446.643 óbitos por todas as causas. O valor informado no artigo corresponde, portanto, a 5,5 vezes o total de óbitos do Capítulo IX nos quatro estados completos, e supera em 45% o total por todas as causas nesses mesmos estados.

Como as regiões metropolitanas representam subconjuntos dos respectivos estados e o Capítulo IX constitui apenas uma fração das causas de morte, o total informado parece incompatível com o universo descrito na seção *Métodos*. Seria importante esclarecer a que população, conjunto de causas e unidade de contagem o valor de 9.357.684 efetivamente se refere - em particular, se ele designa óbitos ou registros de outra natureza.

## A razão observado/esperado não é apresentada

O item de estimativas de excesso de mortalidade define detalhadamente a razão observado/esperado (O/E), e o resumo informa que o excesso de mortalidade foi estimado por esse método, com resultados indicando óbitos em excesso durante eventos específicos.

Não identificamos, no corpo do artigo, a apresentação de nenhum valor de O/E - nem por evento, nem por metrópole, nem de forma agregada. O artigo apresenta uma tabela, com a contagem de ondas de calor por ano e metrópole, e uma figura, com a série temporal de mortalidade; nenhuma das duas traz razões O/E. A seção *Resultados* afirma que a análise da razão de observado *versus* esperado indica aumento durante episódios específicos de onda de calor, sem apresentar as estimativas correspondentes, seus intervalos de confiança ou os testes empregados.

Considerando que a razão O/E constitui o principal indicador utilizado para sustentar a associação entre ondas de calor extremo e excesso de mortalidade, sua apresentação integral contribuiria para a transparência e a reprodutibilidade da análise, permitindo ao leitor avaliar a magnitude do efeito, sua consistência entre os 11 eventos identificados e sua precisão estatística, além de possibilitar comparações com os estudos que empregam a mesma metodologia, inclusive aqueles citados pelos autores como origem do método.

Registramos, ainda, que a equação 4, tal como impressa, parece não corresponder à definição verbal do estimador: o divisor (k − 1) aparece aplicado apenas ao último termo do denominador, e não à soma dos períodos de comparação, como exigiria o cálculo da média descrito no texto. Um esclarecimento sobre a forma correta da equação seria oportuno.

## Achados anunciados sem análise correspondente

O resumo afirma que a mortalidade por causas cardiovasculares coincidiu com os episódios de onda de calor “*particularmente entre os idosos e nas maiores regiões metropolitanas*” (p. 1), e a seção *Resultados* repete a afirmação, referindo-se a áreas metropolitanas de maior densidade populacional. Não identificamos, na seção *Métodos*, estratificação por faixa de idade nem variável de densidade populacional, e não localizamos no artigo resultados correspondentes a essas duas comparações.

De modo semelhante, os *Métodos* informam a realização de uma análise de regressão e de uma análise de correlação entre o EHF e os óbitos diários por hipertensão arterial e isquemia, cujos resultados também não localizamos no texto. Seria útil que essas análises fossem apresentadas, ou que se esclarecesse se foram efetivamente conduzidas.

## Granularidade dos dados e o estimador empregado

A descrição da base de mortalidade especifica dados mensais, obtidos do TabNet, e o resumo refere-se a dados agregados. A subseção *Estatística*, entretanto, menciona a análise de correlação do EHF com os óbitos diários, e a equação da razão O/E é definida a partir dos óbitos observados durante os dias das ondas de calor (eventos de três a seis dias), comparados aos óbitos dos mesmos dias em anos de referência.

Dados agregados por mês não permitem isolar os óbitos ocorridos em dias específicos. Há, portanto, uma aparente incompatibilidade entre a granularidade temporal descrita na seção de dados e a exigida em outras duas passagens do artigo. Um esclarecimento sobre qual granularidade foi efetivamente utilizada, e sobre a origem dos dados diários, caso tenham sido empregados, permitiria avaliar essa etapa da análise e replicá-la.

## Identificação e classificação dos eventos

O texto apresenta a Tabela 1 como contendo as temperaturas médias diárias entre 2014 e 2023, enquanto a tabela traz a contagem de ondas de calor por ano e metrópole. Não localizamos, no artigo, os valores de T95, de EHF85 ou de EHF, de modo que não é possível ao leitor verificar por que determinado período foi classificado como onda de calor extrema.

O texto afirma, além disso, que foram determinados 11 eventos de onda de calor extremo, e a Tabela 1 soma 11 eventos. A enumeração narrativa dos episódios, porém, descreve 12: para Belo Horizonte, em 2021, são citados dois períodos distintos (de 1º a 4 de agosto e de 28 a 30 de agosto), enquanto a Tabela 1 registra um único evento naquela célula.

Como esse conjunto de eventos define a base de toda a análise subsequente, incluindo o cálculo da razão O/E, esclarecimentos sobre a contagem efetiva e sobre os valores do índice que sustentaram a classificação contribuiriam para a reprodutibilidade.

Os pontos aqui apresentados não diminuem a relevância do tema nem o mérito do esforço empreendido. Ao contrário: a caracterização de eventos térmicos extremos em metrópoles brasileiras permanece agenda necessária, e são justamente os estudos que se propõem a essa tarefa que mais se beneficiam de descrição metodológica suficientemente detalhada para permitir sua reprodução independente. Entendemos que esclarecimentos sobre essas questões fortalecerão a consistência do trabalho e facilitarão sua utilização por pesquisadores e gestores envolvidos na vigilância dos impactos das mudanças climáticas sobre a saúde.
